# Advances in the Diagnosis of Pancreatic Cystic Lesions

**DOI:** 10.3390/diagnostics12081779

**Published:** 2022-07-22

**Authors:** Claudia Irina Pușcașu, Mihai Rimbaş, Radu Bogdan Mateescu, Alberto Larghi, Victor Cauni

**Affiliations:** 1 Gastroenterology Department, Colentina Clinical Hospital, 020125 Bucharest, Romania; puscasu_ic@yahoo.com (C.I.P.); bogmateescu@gmail.com (R.B.M.); 2 Department of Internal Medicine, Carol Davila University of Medicine, 050474 Bucharest, Romania; 3 Digestive Endoscopy Unit, Fondazione Policlinico Universitario Agostino Gemelli IRCCS, 00168 Rome, Italy; alberto.larghi@yahoo.it; 4 Urology Department, Colentina Clinical Hospital, 020125 Bucharest, Romania; victorcauni@yahoo.com

**Keywords:** pancreatic cystic lesion, pancreatic cystic neoplasm, intraductal papillary mucinous neoplasm, endoscopic ultrasound, fine-needle aspiration

## Abstract

Pancreatic cystic lesions (PCLs) are a heterogenous group of lesions ranging from benign to malignant. There has been an increase in PCLs prevalence in recent years, mostly due to advances in imaging techniques, increased awareness of their existence and population aging. Reliable discrimination between neoplastic and non-neoplastic cystic lesions is paramount to ensuring adequate treatment and follow-up. Although conventional diagnostic techniques such as ultrasound (US), magnetic resonance imaging (MRI) and computer tomography (CT) can easily identify these lesions, assessing the risk of malignancy is limited. Endoscopic ultrasound (EUS) is superior to cross-sectional imaging in identifying potentially malignant lesions due to its high resolution and better imaging characteristics, and the advantage of allowing for cyst fluid sampling via fine-needle aspiration (FNA). More complex testing, such as cytological and histopathological analysis and biochemical and molecular testing of the aspirated fluid, can ensure an accurate diagnosis.

## 1. Introduction

Pancreatic cystic lesions (PCLs) are a heterogenous group of lesions, ranging from benign to malignant, often diagnosed incidentally in asymptomatic patients undergoing imaging studies. Some cystic lesions, such as intraductal papillary mucinous neoplasms (IPMNs), mucinous cystic neoplasms (MCNs) and cystic pseudopapillary neoplasms, have malignant potential; and early detection of these types of PCLs is paramount to prevention and treatment of pancreatic cancer at an early stage. Most often, PCLs are initially diagnosed by conventional imaging modalities, such as abdominal ultrasound (US), computer tomography (CT) and magnetic resonance imaging (MRI), but clear differentiation between benign and malignant cysts at this stage is often cumbersome. Endoscopic ultrasound (EUS) aids in the diagnosis and differentiation of PCLs by offering a better characterization of the lesions, which can predict malignant risk, and allows for cyst fluid sampling for further analysis via fine needle aspiration (FNA).

## 2. Epidemiology

With the increased use of and improvements in imaging techniques, PCLs are being diagnosed more often and at earlier stages. However, the exact prevalence of PCLs in the general population is difficult to be determined, particularly due to large variations depending on diagnostic modalities. The prevalence of PCLs is higher when MRI is used compared to CT (20% vs. 3%) [1], but it can be as high as 24.3% in autopsy studies [2]. Incidental diagnosis during EUS for non-pancreatic related examinations can be as high as 9.4% [3], and although more accurate for diagnosing and describing these lesions, the lower availability of EUS compared to MRI or CT might account for the lower diagnostic rates. One recent meta-analysis of 17 studies reported a pooled prevalence of PCLs of 8% but with significant heterogeneity across studies due to the different diagnostic tests used and age groups that were included [4].

One of the findings constantly present across studies is the increased prevalence of PCLs with age. Risk factors for development of PCLs include age, race, personal history of acute pancreatitis and family history of pancreatic cancer [5]. The risk of PCLs increases after the age of 40 years, although there are studies describing incidences as high as 9.1% in patients under 40 years [5]. Moreover, older patients tend to have multiple cystic lesions and a higher risk of developing malignancy [6]. One study described a higher prevalence of PCLs in Asians compared to non-Asians, although there were no other differences regarding cyst size or malignant potential between the groups [7]. Personal or family history of pancreatic disease affects the prevalence of PCLs. There are reports of PCLs being up to three times more frequent in individuals with a family history of pancreatic cancer, and PCLs being frequently misdiagnosed as pancreatic neoplasms in patients with a history of acute pancreatitis [8].

## 3. Classification of Pancreatic Cysts

Pancreatic cysts can be classified according to tissue of origin or neoplastic potential (Table 1). Non-neoplastic lesions accounted for up to 80% of PCLs in earlier studies, but improvements in imaging tests have led to a “pandemia” of pancreatic cysts that we face today, most of them being intraductal papillary mucinous neoplasms (IPMNs). However, pancreatic cystic neoplasms’ (PCNs) prevalence tends to increase with age [9]. Among PCNs, the most frequently discovered lesions beside IPMNs are mucinous cystic neoplasms (MCNs), serous cystic neoplasms (SCN) and cystic neuroendocrine neoplasms [10].

## 4. Current Diagnostic Approach

Even if asymptomatic and incidentally discovered, the primary objective is to differentiate a cyst with malignant potential from a non-neoplastic cyst. The initial approach starts with non-invasive tests, such as cross-sectional imaging, which can be diagnostic in most cases. Sampling of the cyst can be performed under endoscopic ultrasound (EUS) guidance, and an array of tests can be performed with the cystic fluid. However, since the pathologist frequently does not have diagnostic material available, a multidisciplinary and multimodal team approach is needed, based on an integrative judgement including imaging findings, cyto- and histopathology data, cyst fluid biochemical and molecular testing. The current management options include no surveillance for clearly proven benign cysts or in non-surgical candidates; surveillance for those with potential for malignant transformation; or surgical resection of a cyst with high risk features for malignancy [11,12,13,14]. The focuses are to identify all malignant lesions that are amenable to curative surgical treatment and lower the number of surgeries performed for those with benign lesions.

**Figure 1:**
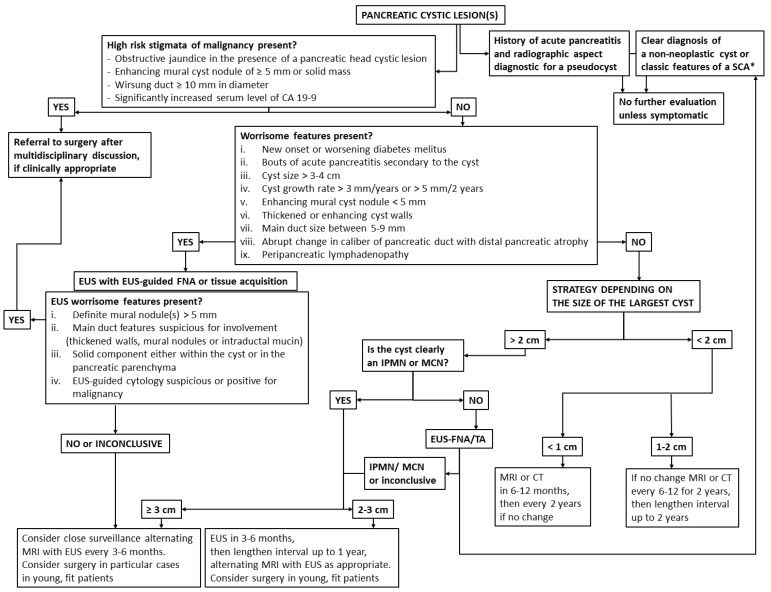
Algorithm of pancreatic cystic lesions (PCLs) diagnosis and management. * Radiographic features diagnostic of a serous cystadenoma are its microcystic appearance and presence of a stellate central scar.

Several guidelines have been published in recent years in an attempt to better guide clinicians to manage these conditions [11,12,13,14]. They all agree that some morphologic features identified in cross-sectional imaging, such as enhancing mural nodules with a size of 5 mm or more, dilation of the main pancreatic duct of more than 10 mm or occurrence of jaundice in the presence of a pancreatic head cystic lesion, represent high risk stigmata and clear indications to operate on a surgically fit patient. For cysts larger than 3–4 cm, the presence of enhancing mural nodules of less than 5 mm or of thickened or enhanced cyst walls; the size of the main pancreatic duct being 5 to 10 mm or an abrupt change in the caliber of main pancreatic duct, with distal pancreatic atrophy; the presence of enlarged lymph nodes; a cyst growth rate of more than 3 mm/year or 5 mm/2 years; new onset diabetes melitus; bouts of acute pancreatitis related to the viscous cyst content; and elevated serum CA19-9 are all considered “worrisome features” prompting EUS evaluation. Confirmation on EUS of a definite enhancing mural nodule ≥5 mm, the presence of main duct features or cytology indicators that indicate or prove malignancy should direct the patient to surgery. For the rest of the definite or suspected neoplastic cysts, the surveillance is adjusted according to their size, for as long as the patient remains a surgical candidate (Figure 1).

Prior to the introduction of clinical guidelines on the management of pancreatic cysts, the surgical resection rates of these lesions were high; almost 40% of the patients who underwent resection for asymptomatic PCLs had benign lesions [15]. Adherence to guidelines is important for the proper management of these cases, as shown in the literature [16,17,18]. For example, in a three-decade study, concordance between preoperative and final histopathological diagnosis increased from 45%, to 68%, to 80% in the new decades, paralleling the use of EUS, EUS-guided cytology, and finally, molecular analysis [19]. Nevertheless, between 10 to 25% of cases referred for surgery still do not have indication for resection, leaving room for improved selection criteria [16,19].

## 5. Differentiating Pancreatic Cystic Lesions from Other Cystic Lesions

Cystic lesions of the abdominal cavity may lead to a diagnostic challenge owing to the overlap in imaging appearance between different entities. Cystic lesions arising from solid organs adjacent to the pancreas can be initially mistaken for PCLs, especially during first-hand investigations such as abdominal ultrasound.

Congenital lesions such as duplication cysts are rare lesions usually arising from the ileum, esophagus or colon, but in rare cases they can arise from the stomach or the duodenum [20]. Lymphangiomas are uncommon benign lesions consisting of enlarged lymphatic vessels, which can develop throughout the gastrointestinal tract [21], with a cyst-like appearance on imaging studies. Cyst formation in a heterotopic pancreas, such as pseudocyst formation or cystic dystrophy, is another rare congenital lesion typically found in the stomach, duodenum or jejunum. The proximity of gastric or duodenal duplication cysts to the pancreas might lead to an initial misdiagnosis of PCLs.

PCLs can arise in genetic syndromes such as autosomal dominant polycystic kidney disease (ADPKD). The prevalence of pancreatic cysts in ADPKD is up to 9% when using US for diagnosis, and patients with the PKD2 gene mutation are five times more likely to develop pancreatic lesions compared to ADPKD patients with the PKD1 mutation [22]. Renal cysts can have extrarenal development, and clear differentiation from a pancreatic cyst is needed, especially to avoid puncturing such a lesion.

Renal, splenic or hepatic pseudocysts can develop as complications after acute pancreatitis, even in the absence of pancreatic pseudocysts. One study presented a case series of eight patients developing renal pseudocysts after acute pancreatitis, half of them maintaining visible communication with the pancreatic duct via EUS [23]. Last but not least, cystic degeneration of solid tumors, due to central necrosis or intratumoral hemorrhage, can be difficult to discriminate from complex true cystic lesions [24].

Imaging studies, and especially EUS, play a central role in differentiating true PCLs from predominantly solid lesions or from cystic lesions of the adjacent organs. A careful inspection for identifying the cyst origin is needed before considering performing more invasive maneuvers, especially if the cysts have contact with the kidneys, since accidentally puncturing some renal cystic lesions, such as perirenal urinomas might determine spillage of urine inside the peritoneum [25].

## 6. The Role of Cross-Sectional Imaging

Not surprisingly, given the advances in cross-sectional imaging and improvements in image quality, most of the pancreatic cystic lesions are currently diagnosed incidentally during such studies performed for other indication [26].

In a meta-analysis on nineteen studies aiming to investigate the role of cross-sectional imaging in PCLs differentiation [27], CT showed a sensitivity of 36.3–71.4% and specificity of 63.9–100% in discriminating benign disease, but with an accuracy for a specific PCL diagnosis of only 39.0–44.7%, proving itself a valuable initial investigation to be used in conjunction with clinical data. In a retrospective study on 80 patients who underwent surgical resection, solid cystic morphology, presence of mural nodules and female gender were associated in multivariate logistic regression analysis with the presence of a premalignant or malignant lesion [28].

MRI/MRCP has very good sensitivity of 91.4–100.0% and specificity of 89.7%, when assessing main pancreatic duct (MPD) communication. In a more recent meta-analysis, MRI had a sensitivity of 76% (95% CI 67% to 84%) and specificity of 80% (95% CI 74% to 85%) for distinguishing benign from malignant lesions, which is similar to the performances of CT scanning [29]. By combining MDCT and MRI, the accuracy of predicting malignancy of a PCL increased from 61% (MDCT alone) to 81% [30]. Lastly, 18-FDG PET/CT had a sensitivity of 57.0–94.0%, a specificity of 65.0–97.0% and an accuracy of 94% in differentiating benign from malignant cysts, which could be useful in equivocal cases [27].

In a study on 86 patients with surgically resected IPMNs (58 benign, 28 malignant), the performances of contrast-enhanced CT and MRI were compared [31]. With both CT and MRI, the presence of an enhancing mural nodule (*p* < 0.001), an abrupt change in the main pancreatic duct caliber (*p* < 0.001), abnormal lymph nodes (*p* = 0.006), a larger main pancreatic duct size (*p* = 0.003) and a fast cyst growth rate (*p* = 0.04) were more common in malignant than benign IPMNs. Irrespective of the modality, the presence of a mural nodule 5 mm or greater across had the highest odds ratio (25 at CT and 29 at MRI) for malignancy. The diagnostic performances of CT (area under the receiver operating characteristic curve, AUROC 0.83) and MRI (AUROC, 0.86) for predicting malignant IPMNs were comparable (*p* = 0.43), showing good intermodality agreement (k = 0.70) [32].

However, the drawbacks of CT and PET/CT imaging include a significant radiation burden, particularly if regular surveillance or follow-up imaging is needed. Furthermore, the presence of mucin inside the cyst can lead to misdiagnosing some lesions as soft tissue rather than cystic content [31]. Moreover, it is difficult to distinguish macrocystic serous cystic neoplasms (SCNs) from other cystic tumors using conventional radiological methods, as evidenced by a study which showed that as much as 51 out of 100 resected and pathologically confirmed SCNs were preoperatively diagnosed as non-SCN lesions [33].

## 7. Endoscopic Ultrasound (EUS)

EUS is rated to have a sensitivity of 88%, specificity of 53% and diagnostic accuracy of 70.4% for neoplastic PCLs [30]. EUS possesses better accuracy for detection of multifocal lesions when compared with CT (47% vs. 13%, *p* < 0.0001) or MRI (58% vs. 34%, *p* < 0.0002) [34].

**Figure 2:**
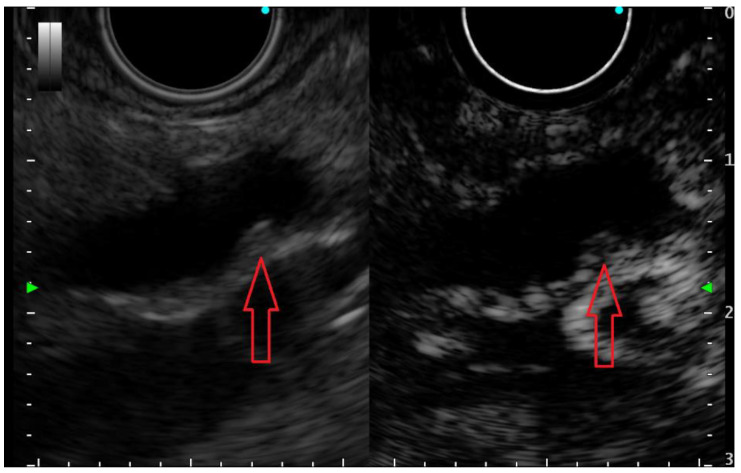
Small (3 mm) mural nodule (red arrow) in a pancreatic cyst—presumably an IPMN, appearing hyperenhanced on contrast-enhanced EUS examination; thus, it is undoubtedly a true mural nodule.

Contrast-enhanced EUS (CE-EUS) remains an important tool in the identification of neoplastic solid components within PCLs, which appear always hyperenhanced (malignant IPMNs and cystic pancreatic neuroendocrine neoplasms), whereas mucus clots and pseudocyst debris appear non-enhanced (Figure 2) [35,36]. In a prospective study on 90 PCL patients, CE-EUS compared favorably with MRI when displaying the inner structure of PCLs and offered advantages over CT in proper cyst classification. In this study, the diagnostic accuracy of CE-EUS for classifying PCLs was higher than that of CT (64.4%, 58/90 vs. 53.6%, 37/69, *p* = 0.017) and equivalent to that of MRI (70.6%, 60/85, *p* = 0.79). However, CE-EUS has been shown to be better for identification of mural nodules compared to CT and MRI (*p* = 0.018 and 0.033, respectively) [18].

EUS can also be used for guiding sampling of the cyst fluid. In a meta-analysis on more than 5000 patients, it was associated with overall morbidity and mortality of 2.66% and 0.19%, respectively [37]. Of the tests that can be performed with the cyst fluid, CEA has been considered the most accurate biochemical marker for differentiating mucinous from non-mucinous pancreatic cysts, the former harboring potential for malignancy transformation. Most importantly however, very low levels of cystic fluid CEA (<5 ng/mL) possess 50% sensitivity and 95% specificity for non-mucinous cyst diagnosis, such as pseudocyst or serous cystadenoma (SCA), and very high levels of this marker (>800 ng/mL) have 48% sensitivity and 98% specificity for mucinous cysts [38,39]. We support the use of these values for proper cyst categorization, rather than the proposed cut-off limit of 192 ng/mL, which has suboptimal discriminating ability (sensitivity of 52–78% and specificity of 63–91%) [12,40].

Moreover, very low cyst fluid glucose levels (≤41 to 50 mg/dL) have been found to be extremely sensitive (88–94%) and accurate (90–95%) for the diagnosis of mucinous pancreatic cysts [41,42,43]. In a systematic review and meta-analysis, glucose level was found to have higher sensitivity and diagnostic accuracy than fluid CEA (91% and 94% vs. 56% and 85%, respectively; *p* < 0.001) in mucinous/non-mucinous cyst differentiation [44]. With the help of a glucometer, these values can be obtained immediately after cyst fluid sampling, and combined with the CEA levels might increase the sensitivity of diagnosing mucinous cysts [45], thereby supporting the use of biochemical tests as the preferred markers, especially in low-volume aspirates.

## 8. Pathology Evaluation

The cytopathological examination of PLCs has a very good specificity (93%) for identifying mucinous cysts with malignant potential. However, its sensitivity is only 54%, mostly because of the very low cellularity of most cysts [46,47,48]. Even if a second EUS-FNA for cytology might be useful in some cases, particularly for diagnosing neuroendocrine pancreatic tumors [49], there is definitely a need for a better test.

Recently, a novel through-the-needle microforceps biopsy (TTNB) was designed for EUS-guided sampling of PCLs walls (Moray™ Microforceps, US Endoscopy, Mentor, OH, USA). A recent meta-analysis showed a technical success of EUS-guided TTNB of 97.1%, a diagnostic yield of 79.6% and a diagnostic accuracy of 82.8% [50], a significant improvement compared to the EUS-guided FNA of the cyst in both diagnostic yield (OR 4.79 (95%CI, 1.52–15.06; *p* = 0.007)) and diagnostic accuracy (OR 8.69 (95%CI, 1.12–67.12; *p* = 0.038)). The severe adverse event rate was 1.1% [51]. Of the non-severe adverse events, intracystic hemorrhage was reported in 5.6% of cases, and acute pancreatitis in 2.4% [51].

In a retrospective multicenter study on 56 patients, the diagnostic yield of EUS-guided TTNB combined with fluid cytology was found to be significantly better than that of EUS-guided FNA needle biopsy of the cyst wall combined with fluid cytology (83.9% vs. 41.6%, *p* < 0.0001) [52]. However, in this study, PCLs clearly connected to the main pancreatic duct represented an exclusion criterion, along with all lesions harboring “worrisome features,” as defined by the International Association of Pancreatology guidelines [13]. Of note, in 12 of the patients, the results of EUS-guided TTNB could be compared with the gold standard of evaluation of the surgically resected piece. The EUS-guided TTNB diagnostic concordance for mucinous lesions (MCN or IPMN) was 91.6%, and its concordance for histologic severity of the lesion was 75% (in three of the 12 lesions, EUS-guided TTNB rated the lesion as less histologically advanced than in reality) [52].

In the most recent prospective single center study, enrolling 101 consecutive patients presenting with a PCL of 15 mm or more and referred for EUS, or a PCL of any size with one or more of either the high risk stigmata or worrisome features, an adverse event (AE) rate of 9.9% was reported [53]. Of these 10 events, 9 were represented by acute pancreatitis. Four of these were considered severe, and there was one fatal outcome. This appears quite an expensive price to pay for a change in clinical management in 11.9% of the cases (about 1 in every 10 patients). As a matter of fact, of these 12 cases in whom EUS-guided TTNB changed the management, 10 had serous cystic neoplasias, in whom surveillance was discontinued. This study, through its prospective nature and relatively large sample size, brought several clarifications. First of all, EUS-guided TTNB is a procedure that could result in severe AEs, especially acute pancreatitis, and for its prevention, the post-ERCP protocol of perioperative Ringer lactate hydration and rectal administration of non-steroidal anti-inflammatory drugs (NSAIDs) probably needs to be applied. Second, considering the possibility of severe adverse events, this procedure needs to be reserved for selected patients who are expected to benefit from an increase in diagnostic yield, for example, patients in whom the surgical indication is unclear. Third, in patients with side-branch IPMNs, EUS-guided TTNB allows for their subclassification into gastric, mixed gastric/intestinal or mixed gastric/pancreatobiliary subtypes, which might be important for the follow-up strategy. Lastly, for specific diagnoses, such as mucinous cystic neoplasms, where the presence of an ovarian type stroma is rarely observed on cytological samples, or for oligocystic type of serous cystic neoplasms, where the cytologic results were inconclusive, the diagnostic adjunct of EUS-guided TTNB seems to be major factor [53].

## 9. Molecular Testing

Recently, cyst fluid molecular analyses that have enabled us to detect differences in gene mutations or protein expression, and in glycoproteomic and metabolomic profiling, have become available.

A retrospective review of 46 consecutive patients showed that adding two simple molecular tests, such as the presence of K-ras point mutation and the allelic imbalance loss-of heterozygosity (LOH), resulted in a change in management in 26 to 28% of cases, according to two independent evaluators. Interestingly, when considering the CEA fluid concentration, those patients with an intermediate fluid CEA level (45–800 ng/mL) or without a fluid CEA concentration available had a more frequent change in management (40% of cases) compared to all others (*p* < 0.05) [54]. However, in a meta-analysis, fluid K-ras evaluation was significantly less accurate than fluid CEA and cytology for the diagnosis of malignant and significant cysts. Sensitivities were 43% and 46% and specificities were 62% and 97%, respectively [55].

Multiple studies have identified molecular markers associated with identification of different types of pancreatic cysts [33,56,57], data which could be used for more accurate diagnosis in these patients in the future, and identification of malignancy transformation by accumulation of genetic alterations [58].

In one prospective study on 130 PCL patients with histopathology available after surgical resection, cyst fluid was analyzed for mutations previously reported in pancreatic cysts (BRAF, CDKN2A, CTNNB1, GNAS, KRAS, NRAS, PIK3CA, RNF43, SMAD4, TP53, VHL), loss of heterozygosity of the tumor suppressor genes (CDKN2A, RNF43, SMAD4, TP53 or VHL) and aneuploidy [59]. Multiple markers associated with different cyst types were identified [60]. More importantly, using a panel of composite molecular markers, predicting of cysts which needed surgical resection (harboring either high-grade displasia or invasive carcinoma) was possible with a sensitivity of 75% and a specificity of 92% [59].

However, when comparing fluid molecular testing with microforceps biopsies from the cyst wall, in a meta-analysis of studies testing for at least four genetic mutations, including KRAS, GNAS, VHL and at least one other genetic mutation characteristic of an aggressive neoplasm (PIK3CA, TP53, SMAD4, PTEN, CDKN2A), on 1206 patients, the diagnostic yields for identification of high-risk cysts, mucinous low-risk cysts and benign cysts were found to be higher for EUS-TTNB than for genetic analysis (73% vs. 54%), but the rates of correctly identified types of cyst were the same (73% vs. 71%) [60].

In a prospective study enrolling 36 lesions (28 classified as mucinous and 6 as non-mucinous), all exons of the following genes were included and sequenced from the cyst fluid by targeted Next-Generation Sequencing (tNGS): AKT1, ALK, APC, BRAF, CDKN2A, CDH1, CTNNB1, DDR2, EGFR, ERBB2, ESR1, FBXW7, FGFR1, FGFR2, FGFR3, FOXL2, GNA11, GNAQ, GNAS, HRAS, IDH1, IDH2, KIT, KRAS, MAP2K1, MET, NOTCH1, NRAS, PDGFRA, PIK3CA, PIK3R1, PTEN, RET, RNF43, ROS1, SMAD4, TGFBR2, TP53 and VHL. Thus, almost complete coverage was obtained. The amount of DNA obtained from sampling was sufficient for molecular analysis in only 69.4% of the pancreatic cysts. Of all these gene analyses, only KRAS and/or GNAS could distinguish mucinous (from non-mucinous) cysts with a sensitivity of 83.3% and a specificity of 60%. None of the other analyzed mutations could reliably differentiate mucinous cysts or to detect malignant cysts with statistical significance [61].

In another study including 102 patients with surgical follow-up, KRAS/GNAS mutations were detected in 56 (100%) IPMNs and 3 (30%) MCNs, and were associated with 89% sensitivity and 100% specificity for mucinous pancreatic cysts [62]. Moreover, by tNGS, the combination of KRAS/GNAS mutations and alterations in TP53/PIK3CA/PTEN had 89% sensitivity and 100% specificity for advanced neoplasia, defined as high-grade dysplasia or invasive adenocarcinoma [63].

In another study testing the mutation allele frequencies (MAFs) of commonly altered genes (BRAF, CDKN2A, CTNNB1, GNAS, RAS, PIK3CA, PTEN, SMAD4, TP53 and VHL) in 318 patients, including 46 in whom surgical resections were performed with histopathological diagnosis available, a sensitivity for the diagnosis of mucinous cysts of 93.3% was found based on the detection of KRAS and/or GNAS gene mutations (*p* = 0.0001). Additional genes provided a marginal improvement in sensitivity but were associated with cyst type (e.g., VHL) and grade (e.g., SMAD4) [62].

Conversely, in another meta-analysis [64] the authors found that if cyst fluid mutational testing for KRAS/GNAS was negative, the probabilities that the patient has an IPMN or a mucinous cystic lesion would be approximately 2% and 8%, respectively [65], a finding with important practical consequences. These data have been incorporated into the last ACG clinical guideline on the diagnosis and management of pancreatic cysts, which specifically recommend molecular testing of the cyst fluid to be considered in cases in which the diagnosis is unclear, and the results are likely to change management, e.g., in the identification of IPMNs and MCNs [11].

Another study focused on proteomic profiling of the cyst fluid in 91 patients by using mass spectrometry. Thirty-three proteins and 32 peptides were found to have different abundances (*p* ≤ 0.05) in different cyst types, and 19 proteins appeared unique to a specific cyst type [64].

Thus, molecular biomarkers indicative of malignancy may seem a very promising tool for the proper identification of cysts needing close follow-up or treatment, but clinicians should be aware of their current diagnostic performance limitations, and of the types of lesions that these tests could be able to correctly identify.

## 10. Needle-Based Confocal Laser Endomicroscopy

The technological progresses over the last decade has made possible the insertion of small real time imaging probes through the lumen of an EUS-FNA needle. Thus, after needle access, evaluation of the cyst walls seemed the next logical step to be performed.

Needle-based confocal laser endomicroscopy (nCLE) is a novel development allowing evaluation of the inner walls of the pancreatic cysts (AQ-Flex nCLE miniprobe, Cellvizio, Mauna Kea Technologies, Paris, France). The feasibility and safety of nCLE in patients with PCLs were confirmed in one international multicenter study and one French multicenter study [66]. nCLE criteria have been developed for the characterization of the most frequent types of PCLs (pseudocysts, mucinous and serous cystadenomas, BD-IPMN and neuroendocrine neoplasms). In a multicenter, adequately powered study, aimed at validation of the previously proposed criteria, of the 206 enrolled patients, 175 (85%) had a conclusive examination. Sensitivity and specificity of 95% and 100% were obtained for the diagnosis of serous cystadenoma and for the differentiation between mucinous vs. non-mucinous PCLs. A sensitivity of 100% and a specificity of 95% were found for diagnosing neuroendocrine neoplasms. Interestingly, the pattern previously described for pseudocysts was observed in three cases other than pseudocysts (two mucinous lesions, one serous cystadenoma), and this criterion was absent in one of the two pseudocysts enrolled in this series. This validation study proved that the criteria need be refined for lesions other than SCA, mucinous lesions and neuroendocrine neoplasms, and also that the procedure had a favorable safety profile (acute pancreatitis reported in 1.3% of cases) [67]. There are, however, more aspects to be cleared up, such as the cost of the procedure, what defines adequate training and the impact on patient management.

In another prospective study of 144 patients with a suspected PCL of 20 mm or more, of whom 65 underwent surgical resection, nCLE was able to differentiate mucinous from non-mucinous PCLs with 98% sensitivity, 94% specificity and 97% accuracy, much better than by using a combination of fluid CEA measurement and fluid cytology (74% sensitivity, 61% specificity and 71% accuracy, *p* < 0.001) [68].

EUS-guided nCLE is thus a minimally invasive procedure improving evaluation of PCLs by routine in addition to standard EUS-FNA, which could positively impact patient management by preventing unnecessary follow-up investigations and/or surgery; however, it is acknowledged that structured training in this technology for competent application is needed [67]. Moreover, the presence of criteria for malignancy (including the presence of mural nodules) was not assessed by the studies performed so far, and thus far there are no defined nCLE criteria for malignancy within a pancreatic cyst.

## 11. Combination of Tests

Given the limitations of all diagnostic tests presented above, expert recommendations are to apply multiple tests in the case of an inconclusive diagnosis. For example, in a meta-analysis on 362 patients investigating the role of fluid cytology and K-ras mutational analysis in the differentiation of mucinous from non-mucinous cysts, the sensitivity and specificity of cytology were 42% and 99% and of K-ras analysis were 39% and 95%; but these increased when the tests were combined to 71% and 88%, respectively [69].

In another study on 122 patients with PCLs, of whom 33 had diagnostic confirmation by histology or surgery, imaging (typical pattern for a serous cyst) or clinical follow-up, the combination of CEA analysis, cytology and viscosity of PCL fluid increased the diagnostic yield for mucinous PCLs to 91%, as compared to 87%, 82% and 84% for the rest of the individual tests, respectively.

In a validation study on 1026 patients using a composite panel of clinical and imaging markers, including patient age and gender, the presence of symptoms such as abdominal pain, jaundice or weight loss, cyst location or multifocality, communication with main pancreatic duct (MPD) and MPD dilation, resulted in sensitivity of 84% and specificity of 81% for correct identification of the cyst type [70]. Applying this set of markers to a cohort of 130 patients enabled identification of cysts that required surgery with a sensitivity of 77% and a specificity of 75% [59]. When molecular markers were added to the composite molecular and clinical markers, the sensitivity to predict which cysts required surgery increased to 89%, but at the expense of specificity, which fell to 69% [59].

Therefore, and as we all do in our current practice, the sum of tests available for a particular patient must be integrated into the diagnostic process and clinical decision making.

## 12. Personalized Approach

A number of studies have focused on patient characteristics rather than cyst features when determining the risk of progression or malignancy of these lesions. A study including 540 patients with BD-IPMN with surveillance performed over a median period of 51.5 months identified progression of the lesion in 130 of them (24.1%) [71]. The outcome measure was appearance of worrisome features or high-risk stigmata, as defined by the International Association of Pancreatology guideline [13], whichever occurred first. The reported probability of progression was 3.7% at 1 year, 23.4% at 5 years and 43.3% at 10 years. However, in this study there were only 15 patients who underwent surgery, of whom seven had malignant histology, and only three patients died from a pancreas-related cause. Apart from the cyst size (HR 2.05, 95% CI 1.44–2.91 for initial cyst diameter larger than 15 mm), body mass index greater than 26.4 kg/m^2^ (HR 1.72, 95% CI 1.19–2.50) and heavy smoking (HR 1.81, 95% CI 1.14–2.86) were identified as independent factors associated with progression risk. In a subgroup analysis on 89 patients, the blood group genotype AA was associated with a higher risk of progression (HR 3.49, 95% CI 1.04–11.71) compared with the OO genotype [72].

In a meta-analysis including 41 studies and 5788 patients, features associated with malignancy in IPMNs were cyst size >3 cm (OR, 62.4; 95% CI, 30.8–126.3), presence of a mural nodule (OR, 9.3; 95% CI, 5.3−16.1), dilatation of the main pancreatic duct (OR, 7.27; 95% CI, 3.0–17.4) and main vs. branch duct IPMN (OR, 4.7; 95% CI, 3.3–6.9) [72]. The authors conclude that not all cyst features should be weighted equally when considering risk of malignancy; cyst size >3 cm is most strongly associated with malignant IPMN. On the other hand, size is not all. In a surgical study, ninety-two percent of the PCLs harboring high-grade dysplasia or adenocarcinoma were smaller than 3 cm in diameter [73].

In a retrospective case–control review study of 338 PCLs, besides cyst features, patient characteristics—age less than 50 years, male sex and 10-pack year smoking history—were significantly associated with a change in management determined by the EUS evaluation, factors based on which referral for EUS evaluation should be also judged [74].

As regards survival, in a multicentric retrospective study including 281 elderly patients with IPMNs (231 with worrisome features and 50 high risk stigmata), with a median follow-up of 51 months, the disease-specific survival (DSS) of the former was as high as 96%, whereas for the latter, there was a 40% risk of IPMN-related death, highlighting on the one hand, non-cancerous mortality in these elderly patients, and on the other, the need for surgical treatment of the latter group harboring high-risk stigmata [75]. In another study including 1800 PCL patients, over a median follow-up of 5.7 years, only 43 (10.7%) of the 402 recorded deaths were PCL related [76].

Adherence to available guidelines is important in order to avoid unnecessary surgery, especially in elderly patients or patients with associated comorbidities [77]. However, all of the above data suggest a personalized strategy for all these patients, i.e., a more patient-centered rather than cyst-centered strategy. Some of the considered risk factors yet need to be incorporated into available guidelines. As regards the decision of adhering to a treatment or surveillance strategy, there is definitely a need for upfront multidisciplinary discussion and inclusion of the patient in the decision making. Thus, regardless of the guideline(s) to follow, the decision to resect versus to follow remains individual, as a personalized approach should be the aim [78].

### The Prospect of Artificial Intelligence in PCL Diagnosis and Management

Artificial intelligence (AI) has been extensively analyzed in medicine and is currently applied for different purposes such as automated image screening, risk stratification and supporting clinical decisions. In the field of gastroenterology, AI has many applications such as liver fibrosis assessment, endoscopic lesion detection and imaging detection of inflammatory lesions [79]. With respect to PCLs, AI has been applied to cross-sectional imaging diagnosis [80], EUS [81] and fluid analysis [82]. The primary objective of AI in evaluating pancreatic cystic lesions is to differentiate between malignant and benign lesions. Although initial results are favorable, practical inclusion of AI in PCLs detection and evaluation is currently limited to clinical studies, and more data are needed for the development of specific diagnosis algorithms.

## 13. Conclusions

Given the increased detection of PCLs, the most accurate and well-grounded evaluation is of the greatest importance, in light of these lesions’ potential for malignant transformation. Misdiagnosis implies either risk of malignant progression in a neoplastic cyst or major surgery for resection of a benign lesion. Advances in EUS evaluation, and the addition of novel biopsy or in vivo histology techniques or cyst fluid molecular biomarkers analysis, provide improved discriminating diagnostic accuracy for pancreatic cystic lesions. However, there is a need for better studies to validate these results and to gradually incorporate these novel tests and techniques into future guidelines.

## Figures and Tables

**Table 1:** Classification of pancreatic cystic lesions (PCLs).

	Non-Neoplastic Cystic Lesions	Neoplastic Cystic Lesions
**Epithelial**	Mucinous non-neoplastic cyst	Intraductal papillary mucinous neoplasm (IPMN)
	Congenital cyst	Mucinous cystic neoplasm(MCN)
	Retention cyst	Serous cystic neoplasm (SCN)
	Peri-ampullary duodenal wall cyst	Cystic neuroendocrine neoplasm
	Endometrial cyst	Acinar-cell cystic neoplasm
	Lymphoepitelial cyst	Serous cystadenocarcinoma
		Solid pseudopapillary neoplasm
		Cystic hamartoma
		Cystic pancreatoblastoma
		Cystic acinar cell carcinoma
		Others
**Non-epithelial**	Pseudocyst	Lymphangioma
	Parasitic cyst	Sarcoma
